# Doxapram versus placebo in preterm newborns: a study protocol for an international double blinded multicentre randomized controlled trial (DOXA-trial)

**DOI:** 10.1186/s13063-023-07683-5

**Published:** 2023-10-10

**Authors:** Jarinda A. Poppe, Robert B. Flint, Anne Smits, Sten P. Willemsen, Kelly K. Storm, Debbie H. Nuytemans, Wes Onland, Marten J. Poley, Willem P. de Boode, Katherine Carkeek, Vincent Cassart, Luc Cornette, Peter H. Dijk, Marieke A. C. Hemels, Isabelle Hermans, Matthias C. Hütten, Dorottya Kelen, Ellen H. M. de Kort, André A. Kroon, Julie Lefevere, Katleen Plaskie, Breanne Stewart, Michiel Voeten, Mirjam M. van Weissenbruch, Olivia Williams, Inge A. Zonnenberg, Thierry Lacaze-Masmonteil, Arjan B.te Pas, Irwin K. M. Reiss, Anton H. van Kaam, Karel Allegaert, G. Jeroen Hutten, Sinno H. P. Simons

**Affiliations:** 1 Department of Neonatal and Pediatric Intensive Care, Division of Neonatology, Erasmus University Medical Center Sophia Children’s Hospital, Room Sk-4113, Wytemaweg 80, 3015 CN Rotterdam, the Netherlands; 2 Department of Hospital Pharmacy, Erasmus University Medical Center, Rotterdam, the Netherlands; 3 Neonatal Intensive Care Unit, University Hospitals Leuven, Leuven, Belgium; 4 Department of Development and Regeneration, KU Leuven, Leuven, Belgium; 5 Department of Biostatistics, Erasmus University Medical Center, Rotterdam, the Netherlands; 6 Department of Neonatology, Emma Children’s Hospital, Amsterdam UMC, Amsterdam, the Netherlands; 7 Amsterdam Reproduction & Development, Amsterdam, the Netherlands; 8 Department of Paediatric Surgery and Intensive Care, Erasmus University Medical Center Sophia Children’s Hospital, Rotterdam, the Netherlands; 9 Institute for Medical Technology Assessment (iMTA), Erasmus University Rotterdam, Rotterdam, the Netherlands; 10 Department of Neonatology, Radboud University Medical Center, Radboud Institute for Health Sciences, Amalia Children’s Hospital, Nijmegen, the Netherlands; 11 Neonatal Intensive Care Unit, Cliniques Universitaires Saint Luc, Brussels, Belgium; 12 Department of Neonatology, Grand hôpital de Charleroi, Charleroi, Belgium; 13 Department Neonatology, AZ St-Jan, Bruges, Belgium; 14 Division of Neonatology, Department of Paediatrics, Beatrix Children’s Hospital, University Medical Centre Groningen, Groningen, the Netherlands; 15 Department of Neonatology, Isala, Zwolle, Zwolle, the Netherlands; 16 Division of Neonatology, Department of Pediatrics, Maastricht University Medical Center, Maastricht, the Netherlands; 17 Neonatal Department, Hôpital Erasme, Université Libre de Bruxelles, Brussels, Belgium; 18 Division of Neonatology, Department of Pediatrics, Máxima Medical Center, Veldhoven, the Netherlands; 19 Neonatology, Vrije Universiteit Brussel (VUB), Universitair Ziekenhuis Brussel (UZ Brussel), Brussels, Belgium; 20 Department of Neonatology, GasthuisZusters Antwerpen, Antwerp, Belgium; 21 Quality Management in Clinical Research (QMCR), University of Alberta, Edmonton, AB Canada; 22 Department of Neonatal Intensive Care, University Hospital Antwerp, Edegem, Belgium; 23 Neonatology and Neonatal Intensive Care Unit, CHIREC-Delta Hospital, Brussels, Belgium; 24 Department of Neonatology, Wilhelmina Children’s Hospital, University Medical Center Utrecht, Utrecht, the Netherlands; 25 Department of Pediatrics, Cumming School of Medicine, University of Calgary, Calgary, AB Canada; 26 Maternal Infant Child & Youth Research Network (MICYRN), Vancouver, Canada; 27 Division of Neonatology, Department of Paediatrics, Willem-Alexander Children’s Hospital, Leiden University Medical Centre, Leiden, the Netherlands; 28 Clinical Pharmacology and Pharmacotherapy, Department of Pharmaceutical and Pharmacological Sciences, KU Leuven, Leuven, Belgium

**Keywords:** Doxapram, Placebo, Randomized controlled trial, Preterm infants, Apnoea of prematurity, Efficacy and safety

## Abstract

**Background:**

Apnoea of prematurity (AOP) is one of the most common diagnoses among preterm infants. AOP often leads to hypoxemia and bradycardia which are associated with an increased risk of death or disability. In addition to caffeine therapy and non-invasive respiratory support, doxapram might be used to reduce hypoxemic episodes and the need for invasive mechanical ventilation in preterm infants, thereby possibly improving their long-term outcome. However, high-quality trials on doxapram are lacking. The DOXA-trial therefore aims to investigate the safety and efficacy of doxapram compared to placebo in reducing the composite outcome of death or severe disability at 18 to 24 months corrected age.

**Methods:**

The DOXA-trial is a double blinded, multicentre, randomized, placebo-controlled trial conducted in the Netherlands, Belgium and Canada. A total of 396 preterm infants with a gestational age below 29 weeks, suffering from AOP unresponsive to non-invasive respiratory support and caffeine will be randomized to receive doxapram therapy or placebo. The primary outcome is death or severe disability, defined as cognitive delay, cerebral palsy, severe hearing loss, or bilateral blindness, at 18–24 months corrected age. Secondary outcomes are short-term neonatal morbidity, including duration of mechanical ventilation, bronchopulmonary dysplasia and necrotising enterocolitis, hospital mortality, adverse effects, pharmacokinetics and cost-effectiveness. Analysis will be on an intention-to-treat principle.

**Discussion:**

Doxapram has the potential to improve neonatal outcomes by improving respiration, but the safety concerns need to be weighed against the potential risks of invasive mechanical ventilation. It is unknown if the use of doxapram improves the long-term outcome. This forms the clinical equipoise of the current trial. This international, multicentre trial will provide the needed high-quality evidence on the efficacy and safety of doxapram in the treatment of AOP in preterm infants.

**Trial registration:**

ClinicalTrials.gov NCT04430790 and EUDRACT 2019-003666-41. Prospectively registered on respectively June and January 2020.

**Supplementary Information:**

The online version contains supplementary material available at 10.1186/s13063-023-07683-5.

## Background

Apnoea of prematurity (AOP), a cessation of breathing, is one of the most common symptoms of an immature breathing system and affects almost 80% of the preterm infants born below a gestational age (GA) of 28 weeks [1]. This poor respiratory control leads to episodes of intermittent hypoxemia and bradycardia. Hypoxemic episodes, especially if prolonged, are associated with increased risks for late death or disability, including motor impairment, cognitive or language delay and risk for visual impairment [2, 3].

Non-invasive respiratory support and caffeine therapy are typically administered to prevent intermittent hypoxemia in preterm infants [4]. Endotracheal intubation and invasive mechanical ventilation can resolve persisting AOP by decreasing the frequency and intensity of hypoxemic episodes, but are associated with an increased risk for bronchopulmonary dysplasia (BPD) and neurodevelopmental impairment [5, 6]. The use of invasive mechanical ventilation is therefore restricted as much as possible in preterm infants. Until now, no consensus has been reached on the optimal treatment strategy for AOP because high-quality trials are lacking.

Doxapram, a respiratory stimulatory analeptic drug, might be considered as an add-on treatment to non-invasive respiratory support and caffeine treatment for persisting AOP in preterm infants. It stimulates the respiratory drive through the brainstem respiratory centre, and the peripheral carotid and aortic chemoreceptors [7]. Unfortunately, well-designed and adequately powered trials on the clinical effect of doxapram are missing. Several observational studies found that treatment with doxapram can be beneficial in reducing the apnoea rate and the number of hypoxemic episodes [8–10]. Doxapram therapy may also be effective in preventing preterm infants from invasive mechanical ventilation [11]. Doxapram may potentially improve neonatal outcomes by preventing preterm infants from the risks, including development of BPD, associated with hypoxemic episodes and invasive ventilation.

On the opposite, concerns on the safety of doxapram are also reported in literature. Described adverse effects include hypertension, irritability, tachycardia, hypokalaemia, gastro-intestinal problems and a prolonged QT interval [12–17]. Some observational studies recommended restricted use of doxapram in preterm infants as they found increased cerebral oxygen consumption, decreased cerebral blood flow, increased electrographic seizure activity (based on amplitude-integrated electroencephalography, aEEG) and less sleep-wake cycling during treatment [18, 19]. The limited studies describing the long-term effects of doxapram are inconclusive. In a cohort study, ten Hove et al. did find a lower risk of the combined outcome death or neurodevelopmental delay in preterm infants treated with doxapram compared to controls that did not receive doxapram after adjusting for confounding factors (OR = 0.54, 95% CI 0.37, 0.78) [20]. On the contrary, Lando et al. [21] found that doxapram treatment may have a negative effect on neurodevelopmental outcome. Although doxapram might reduce AOP and seems effective to avoid invasive ventilation on the short term, its long-term effects are to be revealed.

To our knowledge, no adequately powered, well-designed, randomized placebo-controlled trial has been performed to study the long-term efficacy and safety of doxapram in the clinical setting [10]. In this study, we hypothesize that doxapram is safe and effective in reducing the composite outcome of death or severe disability at 18 to 24 months corrected age as compared to placebo.

## Methods and analysis

### Trial design

The DOXA-Trial is a multicentre, double blinded, randomized, placebo-controlled trial with two arms, designed to evaluate the superiority of doxapram compared with placebo in the treatment of AOP. This study protocol followed the SPIRIT (Standard Protocol Items: Recommendations for Interventional Trials) guidelines [22], whereas the trial will adhere to the CONSORT (Consolidated Standards of Reporting Trials) statement [23].

### Study setting

Patients will be recruited from neonatal intensive care units (NICUs) of the participating centres in the Netherlands, Belgium and Canada. Recruitment started in August 2020 and is expected to continue for 4 years. The DOXA-trial is coordinated by the Neonatology Network Netherlands (N3) for the Dutch centres, the University Hospitals Leuven for the Belgian centres and the Maternal Infant Child and Youth Research Network MICYRN for the Canadian centres. Participation of additional (international) centres during the trial is encouraged.

### Study participants

Patients are eligible for inclusion if the following criteria are met: (1) GA at birth below 29 weeks, (2) postnatal age of at least 120 h, (3) adequately dosed caffeine (max 50% increase above standard daily maintenance dose), (4) non-invasive respiratory support according to the local treatment policy, (5) frequent and/or severe apnoea that require a medical intervention as judged by the attending physician (indication to start doxapram, to provide additional caffeine or to start mechanical ventilation if not included in the trial) and (6) written informed consent of parents or legal representatives. A pragmatic study design is chosen, because we anticipate considerable variation between centres and physicians in the judgment on what frequency and duration of apnoea is considered unacceptable and requires intervention.

Exclusion criteria are as follows: (1) previous use of open label doxapram, (2) use of theophylline to replace doxapram, (3) chromosomal defects, (4) major congenital malformations that compromise lung function resulting in chronic ventilation, or increase the risk of death or adverse neurodevelopmental outcome and (5) palliative care or treatment limitations because of high risk of impaired outcome.

Several conditions, such as sepsis, pneumonia, necrotizing enterocolitis (NEC) and patent ductus arteriosus may present with apnoea and respiratory failure. Distinction from apnoea caused by prematurity is difficult or even impossible. Therefore, the suspicion of one of these diagnoses is not considered an exclusion criterion. However, the diagnosis may result in a decision to primarily intubate the infant and not include the patient in the trial at that time point. This decision is left to the attending physician and medical team. Discontinuing study medication is always allowed if judged necessary by the attending physician and medical team, although discouraged to prevent more open label treatment in the placebo group.

### Pharmacological intervention

Participants will be randomized to receive either doxapram (as hydrochloride, 2 mg/ml in dextrose 5%, 50 mL) or placebo (dextrose 5%, 50 mL). Study medication is manufactured in ready to use 50-mL polypropylene vials and labelled by *Apotheek A15* in Gorinchem, the Netherlands, according to Good Clinical Practice and Good Manufacturing Practice guidelines. Doxapram and placebo fluids are visually identical as well as the labels and the vials, and the syringes in which the study medication will be prepared for blinded administration. Central stock of study medication is stored in the trial pharmacy of Erasmus MC in Rotterdam and dispensed to participating hospitals to supply the local stock.

Therapy is started with a loading dose of 2.0 to 2.5 mg/kg administered intravenously in 10 min, followed by a continuous maintenance dose of 0.5 to 1.0 mg/kg/h via intravenous infusion [17, 24]. The maintenance dosage can be titrated based on the clinical response of the patient; an increase of 0.5 mg/kg/h is recommended after 30–60 min in case of an insufficient effect of the study medication. Increases are allowed up to 2.0 mg/kg/h. If the study medication is effective, clinicians are encouraged to assess the possibility of step-by-step dose reduction of 0.5 mg/kg/h every 12–24 h. Dosage adjustments can also be made if side effects are suspected. Using the same formulation, a switch to gastro-intestinal administration via the nasogastric tube is feasible and allowed [25]. Gastro-enteral administration can be either continuous or intermittently from 4 times daily up to 24 times per day. Oral administration of the study medication requires a 33% higher dose, with a maximum dose of 2.0 mg/kg/h, compared to intravenous administration, due to the oral bioavailability of doxapram (74%) in preterm neonates [26].

**Fig. 1:**
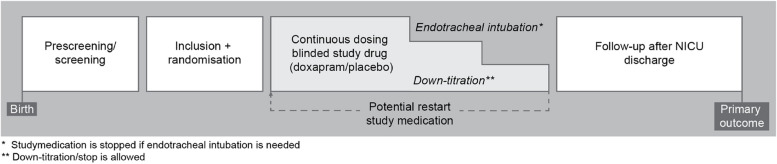
Overview of the study procedures

**Fig. 2:**
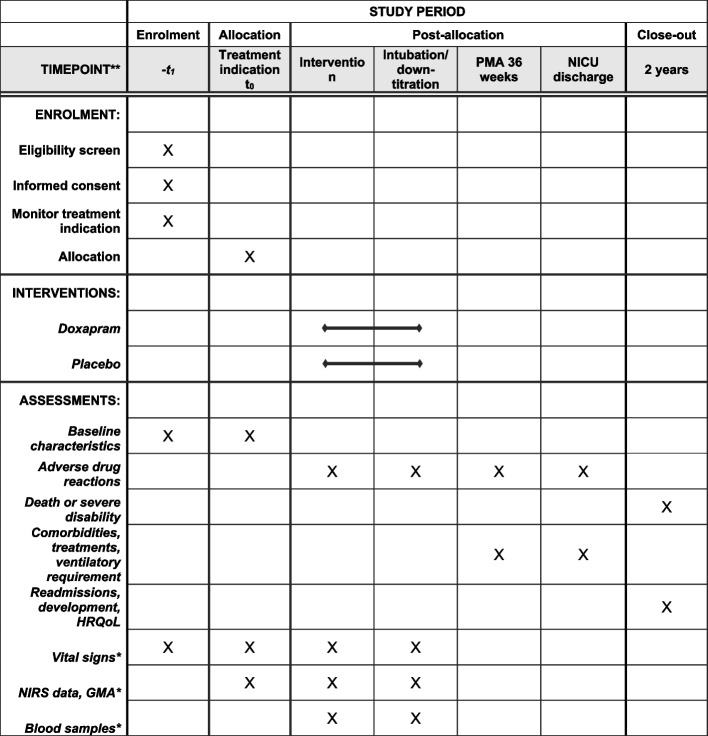
Schedule of enrolment, interventions and assessments

Therapy is continued until the number and duration of the apnoea decrease below the estimated intervention threshold, or until respiratory failure occurs and invasive mechanical ventilation is needed according to the discretion of the attending physician (Figs. 1 and 2). Physicians are allowed to shortly interrupt the continuous administration of study medication in order to administer other (incompatible) medication intravenously. If the study medication is paused for less than 2 h, no additional action is necessary. An additional half-loading dose of the study medication should be administered if the interruption lasts between 2 and 4 h and a standard loading dose if the pause lasts longer than 4 h.

In case of recurrent apnoea needing intervention after down-titration and study medication cessation, the study medication can be restarted according to the same guidelines as the first administration. Open label use of doxapram in participants of this study is prohibited. A patient will remain in the same randomized study group.

#### Primary outcome

The primary outcome is the composite outcome of death or severe disability at 18 to 24 months corrected age.

Severe disability will be defined as having one or more of the following outcomes at the age of 18 to 24 months: cognitive delay, cerebral palsy, severe hearing loss, or bilateral blindness. Cognitive delay will be defined as a Mental Development Index score of less than 85 on the Bayley Scales of Infant and Toddler Development III [27]. Cerebral palsy will be diagnosed if the patient had a non-progressive motor impairment, characterized by abnormal muscle tone and decreased range or control of movements. The level of gross motor function will be determined with the Gross Motor Function Classification System and is defined as severe movement disability if the score is > level 2 [28].

Severe hearing loss will be defined as hearing loss requiring hearing aids and will be measured by audiometry in patients with signs of hearing abnormalities at the corrected age of 18 to 24 months [29]. Hearing will be assumed to be normal in participants with a normal hearing screening after birth and no signs of hearing abnormalities at the corrected age of 18 to 24 months. Severe visual impairment or blindness will be defined as a corrected visual acuity less than 20/200 [30].

#### Secondary outcomes

Secondary outcomes with details on the statistical analyses are summarized in Table 1.

**Table 1:** Summary of the secondary objectives with the statistical analysis and time of measurement

	**Secondary outcomes**	**Details of statistical analysis**^**a**^	**Time of measurement**
**Short-term mortality and comorbidity**	- Bronchopulmonary dysplasia (NICHD criteria) - Death	Logistic regressions will be performed, corrected for GA and centre, for BPD and death separately, and the combined outcome of BPD and death. Survival analysis will be performed for time to death. Cause-specific risk models will be computed to consider death as competing risk for severe disabilities at 18 to 24 months of age.	Postmenstrual age of 36 weeks and hospital discharge
	**Admission:** - Length of stay intensive care - Length of stay hospital	Survival analyses will be performed for time to discharge.	Hospital discharge
	**Respiration:** - Incidence of endotracheal intubations - Number of days on invasive ventilation - Number of days on respiratory support - Number of days with supplemental oxygen - Respiratory complications - Use of postnatal corticosteroids	Logistic regression analysis will be performed for the dichotomous respiratory complications and the use of corticosteroids. Linear regression analysis will be performed for the incidence of endotracheal intubations, and number of days on invasive ventilation, respiratory support, and supplemental oxygen. In both analyses will be corrected for GA and centre. A survival analysis will be performed for the time to intubation.	Hospital discharge
	**Gastro-intestinal:** - Solitary intestinal perforation - Necrotizing enterocolitis > stage 2 (Bell’s criteria) - Feeding problems (days with parental feeding after inclusion) - Body weight - Head circumference	Logistic regression analysis will be performed for SIP, NEC and feeding problems. Linear regression analysis will be performed for body weight and head circumference. Both analyses will be corrected for GA and centre.	Hospital discharge
	**Neurological:** - Intravenous haemorrhage - Clinical seizures - Periventricular leukomalacia	Logistic regression analysis will be performed for all neurological outcomes, corrected for GA and centre.	Hospital discharge
	**Complications:** - Incidence of late-onset sepsis - Meningitis after inclusion - Need for inotropes/ respiratory support	Logistic regression analysis will be performed for all complications, corrected for GA and centre.	Hospital discharge
	- Retinopathy of prematurity	Logistic regression analysis, corrected for GA and centre, will be performed for retinopathy ‘yes’ or ‘no’.	Hospital discharge
	- Hearing loss	Logistic regression analysis, corrected for GA and centre, will be performed for hearing loss ‘yes’ or ‘no’.	Hospital discharge
**Long-term outcomes**	- Readmission since first hospital discharge - Body weight - Body length - Head circumference - Behavioural problems (Child Behavior Checklist) - Cognitive and language development	Logistic regression analysis will be performed for readmissions, behavioural problems, and cognitive and language problems. Linear regression analysis will be performed for body weight, body length, and head circumference. In both analysis will be corrected for GA and centre. Survival analysis will be performed for the time to readmission.	Eighteen to 24 months of corrected age
**Cost-effectiveness**	- (Non-)medical costs during and after hospital admission - Patient outcomes; survival, severe disability, and health-related quality of life (HRQoL)	The total costs per patient and quality-adjusted life years (QALYs) will be assessed. Incremental cost-effectiveness ratios (ICERs) will be calculated, expressed as incremental costs per additional survivor without severe disability at 2 years and incremental costs per QALY gained.	Eighteen to 24 months of corrected age
**Pharmacokinetics**	- Blood samples	Exposure of (keto-) doxapram will be calculated from an existing PK model. The cumulative exposures will be calculated and analysed using a linear regression model, corrected for centre.	During level 3 NICU admission
**Respiratory effects**	- Vital sign data (oxygen saturation, respiratory rate, heart rate, blood pressure) - Ventilation data (ventilation mode, fraction of inspired oxygen, flow)	Vital sign data will be analysed as continuous data. Logistic regression analysis will be performed for all vital sign data, corrected for GA en centre. Mixed model analysis will be performed to take into account repeated measurements. Ventilation duration and cumulative inspired oxygen need and flow will compared using the *t*-test or Mann-Whitney test.	During level 3 NICU admission
**Cerebral effects**	- Cerebral oxygenation (rScO_2_; NIRS-derived) - Transfer function (rScO_2_ as surrogate for cerebral blood flow) - Neurological function (General movement assessment) - Cerebral activity (aEEG or full EEG)	Continuous outcome data will be compared using a two-sided stratified MWU (van Elteren test). For dichotomous data, a stratified *χ*^2^-test will be used. A linear mixed model for longitudinal measures using restricted cubic splines to model the relation between time and outcome will be used.	During level 3 NICU admission

Secondary, continuous outcomes will also be presented as mean and standard deviation if normally distributed, and as medians and interquartile range if not normally distributed. The Kolmogorov-Smirnov test will be performed to assess normality of continuous variables. Secondary, categorical outcomes will be presented as frequency and percentage. The outcomes will be compared between the doxapram and placebo group using the *t*-test or the Mann-Withney test in case of continuous data, and the chi-square test or Fisher’s exact test in case of categorical data

^a^The effect measures of logistic regression models will be presented as the odds ratios together with the 95% confidence intervals based on the profile likelihood. For linear regression models, robust standard errors computed with a sandwich estimator will be used for confidence intervals and significance tests. Effect measures of the Cox proportional hazards model will be presented as (conditional) hazard ratios together with 95% confidence intervals. Significance will be tested using the partial likelihood ratio test

### Secondary short-term outcomes

Secondary outcomes include the respiratory condition of the patient (BPD (NIH 2001 definition [6]), number of days on invasive ventilation, incidence of endotracheal intubation), death (before a postmenstrual age of 36 weeks), duration of NICU and hospital admission, use of postnatal corticosteroids, body weight (at 36 weeks and hospital discharge), solitary intestinal perforation or NEC > stage 2 according to Bell, clinical seizures, intravenous haemorrhage, incidence of late-onset sepsis, meningitis, need for inotropes, retinopathy of prematurity, and an abnormal hearing test at term equivalent age.

### Adverse drug reactions

Adverse drug reactions will be monitored and registered during therapy. For severe events, we refer to the paragraph on safety reporting. Adverse drug reactions will include potassium levels, discomfort or irritability, the occurrence of tachycardia, hypertension, prevalence of convulsions and NEC. Both causality and severity will be evaluated for the possible adverse drug reactions. Causality will be assessed according to Kramer’s algorithm, which can be used to differentiate between possible and probable side effects [31]. The severity of all possible and probable adverse events will be assessed according to the INC Neonatal Adverse Events Scale [32].

### Secondary long-term outcomes

Additional long-term outcomes at 18 to 24 months corrected age will be hospital readmissions, weight, length and head circumference and behavioural problems according to the Child Behavior Checklist [33]. The cognitive and language development will be assessed by the parent-reported PARCA-R questionnaire [34].

Moreover, we will carry out an analysis of cost-effectiveness, including an analysis of health-related quality of life (HRQoL). The potential health and economic gains of doxapram could be substantial due to the low price of doxapram (off-patent) and because it is expected to result in both shorter duration of ventilation, a decrease in length of hospital stay, improved life-long neurological development and less long-term medical needs. We will explore the plausibility of this hypothesis by performing a cost-utility analysis alongside the trial.

#### Sample size calculation

The sample size calculation is based on a cohort study that investigated the long-term neurodevelopmental outcome of preterm infants who received doxapram therapy compared to non-treated matched control patients [20]. This paper described that 30% of the doxapram-treated patients had the composite outcome death or neurodevelopmental delay at 2 years corrected age compared to 50.8% in the non-treated control patients. We therefore consider an absolute decrease of 15% in the composite outcome as clinically relevant, and we aim to reduce the outcome from 50 to 35% with a number needed to treat of around 7 patients. With an alpha of 0.05 and a power over 80%, a sample size of 170 patients is needed per treatment arm when we use a continuity correction. Because we expect approximately 10 patients to be part of twins and a loss to follow-up rate (excluding death) of 10% per treatment arm, the sample size is 198 patients per treatment arm, adding up to a total of 396 patients to be included. Next to the formal consent letter, an animation and flyer are designed providing additional study information to optimize consent and to reach the targeted sample size.

#### Interim analysis and stopping rules

After inclusion of 80 (20%), 200 (50%) and 300 (75%) patients, an interim analysis will be performed to protect the safety, validity and credibility of the trial. There will be no stopping rules for futility. Patient inclusion will be stopped, however, if neonatal mortality is at least 10% higher (absolute difference) in either the placebo arm or the doxapram arm, and if this difference is statistically significant. A difference is assumed statistically significant if a two-sided *p*-value is less than 0.05, based on Fisher’s exact test. The confidence interval for the risk difference will be based on the method of Newcombe [35]. These analyses will not be adjusted for covariates. A reduced set of baseline variables, including at least gestational age and sex, and outcomes, including at least death, BPD and the composite outcome death or severe disability (where already available), will also be studied at the time of the interim analysis.

#### Randomization and blinding

Participants will be randomly allocated in a 1:1 ratio, stratified according to study centre and gestational age (<26 weeks or ≥26 weeks), and using random permutated blocks. Multiple birth infants will be randomized independently, unless parents or legal representatives explicitly request that siblings are to be allocated to the same treatment arm. Randomization and drug supply management is performed via an electronic online platform (ALEA®, FormsVision BV, Abcoude, the Netherlands).

Once the participant has been randomized, a study medication kit will be allocated. All treatment kits will be visually identical and contain either doxapram or placebo. The clinical trial centre and trial pharmacy of the Erasmus Medical Centre will manage the randomization list. The parents, researchers and medical team will have no access to the randomization list. Unblinding is only performed by an independent safety officer in emergency situations where knowledge of the study drug is considered absolutely necessary for the clinical management of the included patient.

#### Statistical analysis

### Primary outcome analyses

Baseline patient and clinical characteristics will be collected. Continuous variables will be presented as mean and standard deviation if normally distributed, and as medians and interquartile range if not normally distributed. The Kolmogorov-Smirnov test will be performed to assess normality of continuous variables. Categorical variables will be presented as frequencies and percentage. A difference with a *p* <0.05 is set as statistically significant.

Primarily, intention-to-treat analyses will be performed. Per-protocol and as-treated analyses will be performed secondarily if needed. The conditional odds ratio will be calculated from a multivariable logistic regression model, adjusted for the stratification factors gestational age and centre, to assess the treatment effect of doxapram on the primary outcome. The 95% confidence interval of the conditional odds ratio will be based on the profile likelihood and a *p*-value will be based on the likelihood ratio test. Hospitals with fewer than ten participants with the composite primary outcome at 18 to 24 months corrected age will be combined in this analysis, to ensure that the parameter estimates converge and increase stability. Parents of twins are able to choose whether they prefer randomization of both infants separately, or in the same treatment arm. In both cases, twins will be analysed as individuals ignoring the correlation between them [36].

The average absolute risk reduction will be estimated from the logistic regression model using the method of standardization to calculate the risk of an adverse outcome in both study arms by taking the difference. The 95% confidence interval for this estimate will be calculated using a bootstrap with 5000 replicates and will be based on the quantiles of the effect estimates across the bootstrap samples. The number needed to treat will be derived from the average risk difference by inverting the number. The point estimate and confidence interval for the relative risk reduction will also be calculated in a similar way as for the average absolute risk reduction, but now taking the quotient instead of difference between the estimates in both arms.

Additionally, the effect of doxapram on the primary outcome will be adjusted for important risk factors for neurodevelopmental impairment, based on the literature and our clinical experience. A multivariate logistic regression model will be computed including the independent variables small for gestational age, sex, and intraventricular haemorrhage in addition to the stratification variables [5, 37, 38]. A complete case analysis will be conducted if less than 10% of the randomized patients has missing data. In case of a higher rate of missing data, multiple imputation with sensitivity analyses will be conducted.

### Secondary outcome analyses

Logistic and linear regression analyses will be performed for dichotomous and continuous secondary outcomes, respectively. Regression analyses will be adjusted for GA and admitted centre. Survival analyses using Cox proportional hazards regression will be performed to investigate the relations between the outcomes time to intubation, time to discharge, and time to death. Cause-specific risk models will be computed to consider death as competing risk for severe disabilities. This is done by estimating cumulative incidence functions using the Fine and Grey model [39].

### Cost-effectiveness analysis

Using the technique of cost-utility analysis (CUA), costs and patient outcomes will be compared between the doxapram and placebo arm. The time horizon will be the 18 to 24 months follow-up period of the trial. Both medical and non-medical costs will be analysed, following recommended methods for economic evaluations and costing studies in healthcare [40, 41]. Medical costs will include both costs during hospital admission and costs after hospital discharge (e.g., costs of visits to the outpatient department, physical therapy, speech therapy, visual and hearing aids). Resource consumption for all these items will be derived from electronic databases and from questionnaires, based on the Medical Consumption Questionnaire of the Institute for Medical Technology Assessment [42]. Non-medical costs related to severe disability, including costs of social care/day care, grade retention, and special education, will be calculated.

Regarding the patient outcomes, the CUA will look at survival, severe disability (as defined above), and HRQoL. HRQoL will be measured using the Child Health Utility 9D (CHU9D) instrument [43, 44]. Based on data on survival and responses to the CHU9D, quality-adjusted life years (QALYs) will be calculated. Finally, incremental cost-effectiveness ratios (ICERs) will be calculated, expressed as incremental costs per additional survivor without severe disability at 18 to 24 months corrected age and incremental costs per QALY gained.

#### Currently planned sub-studies

For all sub-studies, the blinding of the patients for the study team and researchers will be maintained during the project.

### Vital sign data analyses

Some of the participating centres will collect continuous high frequency monitor data that will be used to analyse the oxygenation in infants receiving either placebo or doxapram. Data on oxygen saturation (SpO_2_), heart rate and respiratory rate will be extracted from bedside monitors where possible. These measurements are part of the standard of care and will be performed without additional burden for participating infants. The vital sign data will be used to investigate the response to doxapram therapy compared to placebo, to identify responders and non-responders to doxapram therapy, and to explore the possibilities of real-time therapy evaluation.

### Neuromonitoring

The impact of doxapram on cerebral oxygenation, cerebral autoregulatory capacity (CAR), cerebral activity and neurological function will be investigated in some of the participating centres. Data collection on brain oxygenation, measured by Near Infra-Red Spectroscopy, includes regional cerebral oxygen saturation (rScO2), cerebral fractional tissue oxygen extraction (cFTOE) and transfer function (TF) gain. Cerebral activity will be assessed by multichannel video-EEG or aEEG. In addition, the neurological function will be assessed using the General Movement Assessment (GMA).

### Pharmacokinetic analysis

We aim to validate the previously reported population pharmacokinetic model of intravenously and gastro-enterally administered doxapram, and its active metabolite keto-doxapram, in preterm infants [26]. Plasma concentrations of doxapram and keto-doxapram will be quantified using an UPLC-MS/MS assay [45]. For this purpose, scavenged samples will be used and/or additional blood samples will be collected during routine blood sampling with a maximum of three samples of 0.1 ml per patient. The plasma concentrations will be quantified in some of the participants (*N*=50) from two participating centres. Because only half of these patients will receive doxapram we will also evaluate the caffeine levels (used as a standard administration in all patients). Caffeine exposure will be compared between the two treatment groups.

### Further sub-studies

Ideas for further sub-studies or analyses are welcome and will be discussed within the steering committee for costs, feasibility and scientific value.

## Ethics and dissemination

The study will be conducted according to the principles of the Declaration of Helsinki [46] and to the Dutch Medical Research Involving Human Subjects Act (WMO) and the Belgian and Canadian Law on patients’ right. Prior to randomization, written informed consent will be obtained according to the national guidelines from both, or at least one of the parents (not allowed in the Netherlands) or legal representatives. The informed consent form is added to the supplements, the additional participant information materials are available from the corresponding author on request. The local study team will provide information on the study for parents or legal representatives of preterm infants born before 29 weeks of GA who may develop an indication for doxapram therapy as soon as possible to allow for sufficient time to consider participation. Extended informed consent will be obtained in the subset of participants for specific sub-studies. The study is approved by the medical ethics committee of the Erasmus University Medical Centre (MEC-2020-0078), the Ethics Committee Research UZ/KU Leuven (S63834) and Health Canada (255525). All substantial amendments to the protocol will be notified to the medical ethics committees and all relevant parties once approval is acquired. Participants can leave the study at any time with no obligation to disclose a reason and without any consequences. The investigator can decide to withdraw a patient from the study for urgent medical reasons. Patients who are withdrawn from the study will be treated according to the standard of care guidelines. As long as parents provide consent, all patients will be followed up including neurodevelopmental outcome assessment in the outpatient clinic. The study sponsors, with the Erasmus University Medical Center as main sponsor for The Netherlands and Belgium and MICYRN (Mother Infant Child Youth Research Network) as sub-sponsor for Canada, have insurances that provide cover for damage to research participants through injury or death caused by the study.

## Data collection and management

Patient data will be collected by the local study team and stored in an electronic database (Castor EDC, Cewit B.V.) according to Good Clinical Practice guidelines. Security is guaranteed with login names, login codes and encrypted data transfer. An experienced data manager will check the database for completeness, consistency and plausibility. The saved data will be coded, and only the local principal investigator, the study team and the monitor have access to the source data. An independent monitor will review the study procedures in each participating centre.

## Dissemination

The study results will be submitted for publication in a peer-reviewed journal and presented at international conferences.

## Oversight and monitoring

An independent Data Safety Monitoring Board (DSMB), including experienced neonatologists, a pharmacologist and a statistician, will monitor the study on safety aspects and will provide recommendations regarding continuing or stopping the trial when 20, 50 and 75% of the anticipated outcome data are available. If the DSMB recommends modification or cessation of the study protocol, this will be discussed with a Trial Steering Committee, who will make the decision. The Trial Steering Committee includes experienced neonatologists as well as network and department directors who will monitor and supervise the progress of the trial. A patient involvement group, Create4Care, supports the trial and has an advisory role in the study. The patient group was involved from the start of the study and can support decisions on modification or cessation of the study protocol.

## Safety reporting

All adverse events reported by the parents of the participant, the investigator or the medical team will be recorded, according to the severity scale of Salaets et al. [32] with a distinction between suspected adverse events, unexpected suspected adverse events and context-specific adverse events. This study population (critically ill preterm infants) has a high risk of serious complications (so-called ‘context-specific serious adverse events (SAEs)’), which are inherent to their vulnerable condition and unrelated to the intervention studied here. These complications are included as primary and secondary outcomes of this study and are recorded in the Case Report Form. This documentation will include the date of diagnosis before or after randomization, classification/grading of the complication and type of action taken (if any). Since these complications are highly interrelated and of longitudinal character, it is impossible to indicate an exact date for the resolution or stabilization of each specific complication. Therefore, we will use the date of discharge from the NICU as the ‘end date’ of the complication.

Suspected unexpected serious adverse reactions (SUSARs) and SAEs will be reported to the sponsor by the local principal investigator within 48 h. The sponsor will report all SUSARs and SAEs (in the Netherlands) to the medical ethical committee of the Erasmus University Medical Centre and to the Central Committee on Research Involving Human Subjects. SUSARs will also be reported to Eudravigilance databank and to the Medicines Evaluation Board [47], and an overview of the SUSARs will be submitted every half year to the medical ethical committee. A safety report including all SUSARs and SAEs will be provided to the medical ethics committee and central committee and Health Canada annually. Parents of participating patients will be asked to report readmissions or other potential SAEs between hospital discharge and 18 to 24 months corrected age.

A special alert procedure will be used in case of the occurrence of a SUSAR (death during administration of the study medication) and NEC with a Bell stage of two or higher during or within 48 h after study medication cessation. These events will be brought directly to the attention of the principal investigator. If necessary, the Steering Committee will alert the DSMB, and the Steering Committee will provide a summary report after every ten alerts to the DSMB.

## Discussion

AOP remains one of the most common diagnoses in preterm infants that can result in intermittent hypoxemia. It is important to protect infants from these periods of hypoxemia as they are associated with an increased risk for adverse long-term outcomes [2, 3]. Doxapram is frequently used off-label in some European countries as an add-on therapy to caffeine and non-invasive respiratory support, aiming to avoid invasive mechanical ventilation in the treatment of persisting apnoea. However, sufficient evidence on the safety and efficacy of doxapram is lacking. Concerns of impact on cerebral function and brain development may hamper the healthy development of doxapram-treated premature infants [18, 19]. Previously, a comparable placebo-controlled trial with caffeine therapy was conducted, resulting in a reduced rate of BPD and an improved rate of survival without neurodevelopmental disability in the caffeine group [4, 48]. Doxapram has the potential to improve neonatal outcomes further [20], but the safety concerns need to be weighed against these potential benefits and the suspected risks of invasive mechanical ventilation. Which of these two is best on the long-term is unknown and forms the clinical equipoise of the current trial. This international, multicentre trial will provide the needed high-quality evidence on the efficacy and safety of doxapram in the treatment of AOP in preterm infants.

Doxapram is at present only registered in children ≥12 years and adults for the treatment of post anaesthetic respiratory depression, where its use is very limited [49]. It is however also suggested to be effective in reducing the number of apnoea and desaturations in preterm infants [8, 9, 50], although this effect is not observed in every infant treated with doxapram [51]. Decades ago, small randomized controlled trials compared doxapram with either placebo or theophylline without evaluating the long-term effects [52–55]. An observational study found a higher cumulative doxapram dosage in preterm infants with neurodevelopmental impairment compared to matched controls with a normal mental development, although this association could be confounded by indication [56]. Another observational study found no increased risk of the combined outcome death or neurodevelopmental delay, and even suggests a positive effect of doxapram [20]. Participants of the trial will be closely monitored, as no firm conclusions on efficacy and safety can be drawn.

A limitation of this trial is that the potential direct respiratory effect of the study medication cannot be blinded, although the assessor of the primary outcome will be blinded for the allocated treatment arm. Another challenge is the absence of uniform criteria to evaluate when the frequency and duration of apnoea are considered unacceptable and an intervention is needed. Poets et al. developed an interesting apnoea score to assess the severity of apnoea, but this score is not validated yet and is mostly a reflection of current clinical practice [57]. The rate of apnoea and hypoxic events can also fluctuate largely within and between patients, and detection can be difficult [51, 58]. We therefore chose for a more pragmatic design with less strict inclusion criteria. The decisions to start doxapram in routine clinical care, to adjust its dosage, or to stop doxapram therapy are made by the attending physicians. Enrolment of a participant in the study is done according to the existing clinical protocols and can vary between centres and physicians, as does the dosing and duration of study drug therapy. This pragmatic strategy was chosen to make the trial clinically feasible, relevant and to increase generalizability to other centres. Randomization of participants is stratified per centre, and we will adjust for this stratification in the analysis. The respiratory status before starting doxapram will be evaluated in a subset of the participants using continuous monitoring data. With these data, we aim to find more objective parameters and criteria for the indication to start doxapram therapy in future patients.

This trial will provide knowledge to improve the optimal treatment of preterm infants suffering from AOP. We will evaluate the safety and efficacy of doxapram in reducing death and severe disability in the first years of life. If doxapram is safe and effective, the treatment could be implemented in more NICUs worldwide. The placebo design may also provide the opportunity to identify patients who will benefit from doxapram therapy and those who will not. Evaluation of the exposure to doxapram and keto-doxapram will enable us to optimize dosing in future individual patients. An improved, more individualized AOP treatment policy will likely prevent patients from periods of hypoxemia and unnecessary treatment risks. Overall, this randomized, placebo-controlled trial aims to improve long-term patient outcomes.

## Trial status

Participant recruitment started in June 2020 and is expected to be completed by May 2025. The current protocol is version 10.0 as of May17, 2023.

### Supplementary Information

**Additional file 1.**

## Data Availability

Data collected from participants will be available to researchers upon reasonable request. Requests should be directed to the principal investigator (s.simons@erasmusmc.nl).

## Abbreviations

AOP: Apnoea of prematurity
BPD: Bronchopulmonary dysplasia
CAR: Cerebral autoregulatory capacity
cFTOE: Cerebral fractional tissue oxygen extraction
CHU9D: Child Health Utility 9D
CONSORT: Consolidated Standards of Reporting Trials.
CUA: Cost-utility analysis
DSMB: Data safety monitoring board
HRQoL: Health-related quality of life
GMA: General Movement Assessment
NEC: Necrotizing enterocolitis
NICU: Neonatal intensive care unit
QALY: Quality-adjusted life years
rScO_2_: Regional cerebral oxygen saturation
SAE: Serious adverse events
SPIRIT: Standard Protocol Items: Recommendations for Interventional Trials
SpO_2_: Peripheral oxygen saturation
SUSAR: Suspected unexpected serious adverse reactions
